# New transgenic mouse models enabling pan-hematopoietic or selective hematopoietic stem cell depletion in vivo

**DOI:** 10.1038/s41598-022-07041-6

**Published:** 2022-02-24

**Authors:** Alessandra Rodriguez y Baena, Smrithi Rajendiran, Bryce A. Manso, Jana Krietsch, Scott W. Boyer, Jessica Kirschmann, E. Camilla Forsberg

**Affiliations:** 1 Institute for the Biology of Stem Cells, University of California-Santa Cruz, Santa Cruz, CA 95064 USA; 2 Program in Biomedical Sciences and Engineering, Department of Molecular, Cell, and Developmental Biology, University of California-Santa Cruz, Santa Cruz, CA 95064 USA; 3 Biomolecular Engineering, University of California-Santa Cruz, Santa Cruz, CA 95064 USA

**Keywords:** Cell biology, Stem cells

## Abstract

Hematopoietic stem cell (HSC) multipotency and self-renewal are typically defined through serial transplantation experiments. Host conditioning is necessary for robust HSC engraftment, likely by reducing immune-mediated rejection and by clearing limited HSC niche space. Because irradiation of the recipient mouse is non-specific and broadly damaging, there is a need to develop alternative models to study HSC performance at steady-state and in the absence of radiation-induced stress. We have generated and characterized two new mouse models where either all hematopoietic cells or only HSCs can be specifically induced to die in vivo or in vitro. Hematopoietic-specific Vav1-mediated expression of a loxP-flanked diphtheria-toxin receptor (DTR) renders all hematopoietic cells sensitive to diphtheria toxin (DT) in “Vav-DTR” mice. Crossing these mice to Flk2-Cre mice results in “HSC-DTR” mice which exhibit HSC-selective DT sensitivity. We demonstrate robust, rapid, and highly selective cell ablation in these models. These new mouse models provide a platform to test whether HSCs are required for long-term hematopoiesis in vivo, for understanding the mechanisms regulating HSC engraftment, and interrogating in vivo hematopoietic differentiation pathways and mechanisms regulating hematopoietic homeostasis.

## Introduction

Permanent or conditional ablation of targeted cell populations has been widely used as a strategy to investigate cell function in vivo. This has been accomplished in a variety of ways, ranging from broadly acting, non-specific targeting to tissue- and cell type-specific approaches^1–5^. Whole body exposure to radiation followed by transplantation has long served as the “gold standard” for understanding the hematopoietic system^6^. Because radiation is non-specific, broadly damaging, and induces a multitude of potentially confounding responses^7–10^, there is a clear need for complementary and more targeted approaches to specifically and efficiently eliminate specific cell types.

One powerful conditioning approach for specific cell ablation is to employ the cytotoxic diphtheria toxin (DT) system where mice are engineered to express either the active A subunit of DT (DT-A) or the human diphtheria toxin receptor (DTR) in a cell type-specific and/or inducible manner. The human DTR (also known as epithelial growth factor receptor, EGFR) is particularly useful in the murine system as DT specifically binds to the human, but not murine, homolog. Therefore, when extracellular DT is administered, only the cells expressing human DTR will be killed, vastly improving specificity^11–13^. Upon induced expression or receptor-mediated endocytic entry into the cytoplasm, DT-A catalyzes the inactivation of elongation factor-2, halting protein synthesis and inducing apoptosis. Therefore, only the cells containing DT-A will be ablated^12,14–16^. Importantly, DT-A toxicity is exceedingly efficient as one molecule in the cytosol is sufficient to induce cell death^16^. The substantial toxicity of DT-A and human DTR specificity results in a combinatorial ablation system that is highly sensitive and efficient.

Here, we generated and characterized two mouse strains with either pan-hematopoietic or hematopoietic stem cell (HSC)-selective DT sensitivity. These two new mouse models enable hematopoietic cell ablation that is magnitudes more specific than currently used strategies such as irradiation and chemotherapy. Thus, they provide a new radiation-independent system that opens new avenues for understanding the mechanisms regulating HSC biology.

## Results

### A novel mouse model for pan-hematopoietic expression of DTR

**Figure 1:**
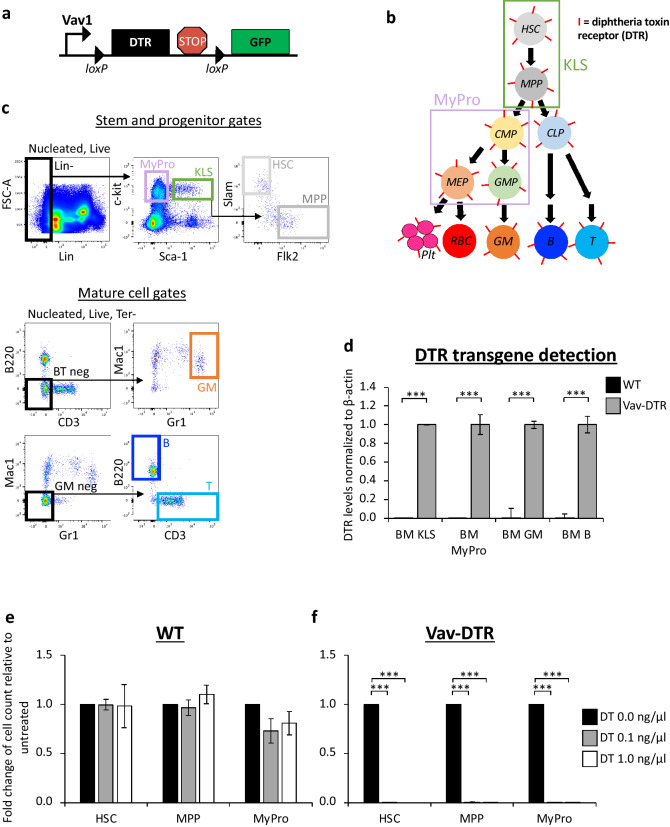
Hematopoietic cells from Vav-DTR mice were sensitive to DT in vitro. (**a**) Schematic diagram of the Vav-DTR transgene construct. (**b**) Simplified model of the hematopoietic tree. All hematopoietic cells that express Vav1 should express DTR (shown as a “I”-shaped surface receptor). (**c**) Representative flow cytometry plots of the main populations analyzed: myeloid progenitors (MyPro), ckit^+^ Lineage^- ^Sca1^+^ (KLS), hematopoietic stem cells (HSC), multipotent progenitor cells (MPP), granulocyte-myelomonocytic (GM), B, and T cells. Pre-gates are shown above the plots. (**d**) Quantitative PCR analysis of BM KLS, MyPro, GM, and B cell populations sorted from WT and Vav-DTR mice detected the DTR transgene only in Vav-DTR mice. Bar graph indicates the relative levels of DTR transgene in cells isolated from WT (black bar) or Vav-DTR mice (gray bar). β-Actin was used to normalize expression levels. N = 3 mice. Error bars indicate SEM, ***p < 0.001 (Student’s t test). (**e**) HSCs, MPPs, and MyPros sorted from WT mice remained unaffected in vitro 7 days after diphtheria toxin (DT) exposure. (**f**) While Vav-DTR cells were drastically depleted. Bar graphs indicate the fold change in cell number relative to untreated (black bar, DT 0.0 ng/µL) after a 7-day 0.1 ng/µL (gray bar) and 1.0 ng/µL (white bar) DT treatment. N = 2 (Vav-DTR) and N = 3 (WT) independent experiments. Error bars indicate SEM, ***p < 0.001 (One-way ANOVA with Dunnett’s post-hoc test). *BM* bone marrow, *HSC* hematopoietic stem cell, *MPP* multipotent progenitor, *CMP* common myeloid progenitor, *CLP* common lymphoid progenitor, *MEP* megakaryocyte-erythroid progenitor, *GMP* granulocyte–macrophage progenitor, *plt* platelet, *RBC* red blood cell, *GM* granulocyte/macrophage, *B* B cell, *T* T cell, *KLS* ckit^+^Lin^−^Sca1^+^ cells include HSCs and MPPs, *MyPro* myeloid progenitors are c-kit^+^Lin^−^Sca1^−^ cells include CMPs, MEPs, and GMPs, *BM* bone marrow.

We sought to generate a novel transgenic mouse line with pan-hematopoietic expression of the human DTR for targeted depletion of all hematopoietic cells. We used the murine regulatory elements of the *Vav1* gene, which is highly and exclusively expressed throughout the hematopoietic system^17–22^ to drive expression of DTR in the “Vav-DTR” mice. Pronuclear injection of the Vav-DTR construct (Fig. 1a) into C57BL/6 zygotes resulted in several Vav-DTR founders with confirmed germline transmission. In this model, DTR would be expressed in all hematopoietic cells, except for red blood cells (RBC, Fig. 1b), similar to our previously published Vav-GFP mouse model^20^. The GFP in the construct would not be expressed unless the Vav-DTR mice contained active Cre-recombinase (Fig. 1a). Presence of the DTR transgene was confirmed to be specific to various hematopoietic cells from Vav-DTR mice and absent in cells from wild type (WT) mice (Fig. 1c,d), as expected.

### In vitro and in vivo depletion of hematopoietic cells is highly specific in Vav-DTR mice

To investigate the functional expression of DTR and specificity of DT sensitivity of hematopoietic cells in vitro, hematopoietic stem and progenitor cell (HSPC) populations were isolated from the bone marrow (BM) of Vav-DTR mice and treated with DT in culture. Independent of the dose, DT did not affect WT cells (Fig. 1e), but very efficiently and significantly depleted HSCs, multipotent progenitors (MPPs), and myeloid progenitors (MyPros) from Vav-DTR mice (Fig. 1f).

**Figure 2:**
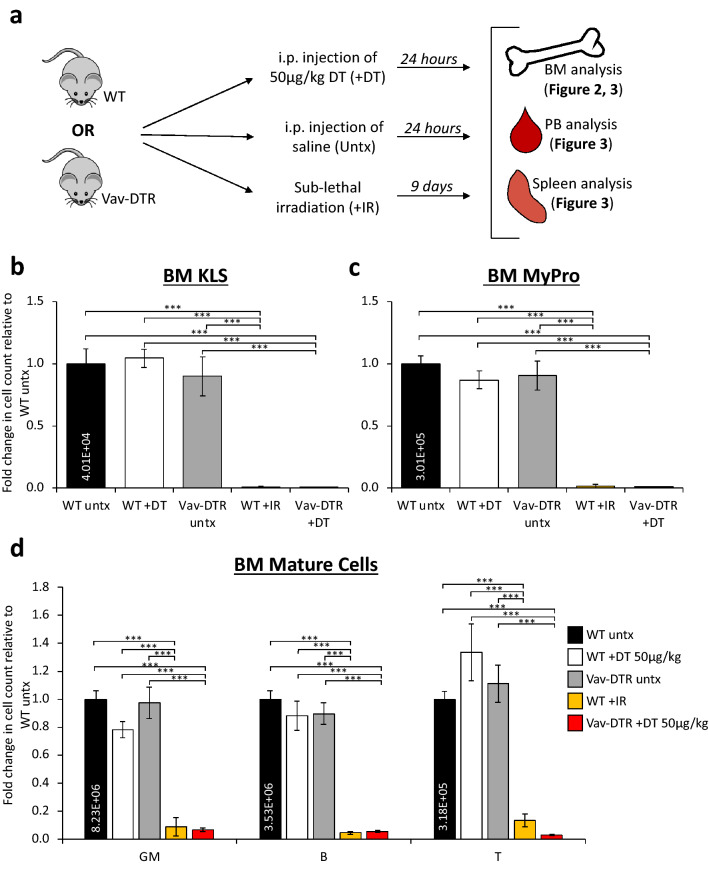
HSPCs and mature cells from the BM of Vav-DTR mice are depleted by DT in vivo. (**a**) Schematic of experimental design. WT and Vav-DTR mice received an i.p. injection of 50 µg/kg of DT 24 h prior to takedown for BM, PB, and spleen analysis. These data were compared to WT and Vav-DTR mice treated with a control saline injection (untreated), and WT mice that were sub-lethally irradiated 9 days prior to takedown. DT depleted KLS (**b**), MyPro (**c**) and mature blood cells (**d**) in the BM of Vav-DTR mice (red bar), similar to 9-days post sublethal (750 rads) irradiation (IR, yellow bar; positive control). WT mice were unaffected by DT treatment (white bars), harboring similar cell numbers to untreated WT mice (black bar; negative control), and untreated Vav-DTR mice (gray bars). The numbers in the black bar represent absolute cell count in the BM. Bar graphs indicate the fold change in cell number relative to untreated WT mice. N = 4–9 mice in at least three independent experiments. Error bars indicate SEM, ***p < 0.001 (One-way ANOVA with Tukey post-hoc test). *HSPCs* hematopoietic stem and progenitor cells, *i.p*. intraperitoneal, *PB* peripheral blood, *Untx* untreated, *IR* irradiated.

We then determined the ability of DT to exclusively deplete hematopoietic cells in vivo, and if the degree of ablation was comparable to irradiation, the most commonly used regimen for ablation of HSPCs from the BM^3,23–25^. To achieve this, we compared cell numbers in the BM and peripheral blood (PB) of WT and Vav-DTR mice 24 h post-treatment with a high dose of DT or saline, or 9 days post sub-lethal irradiation (Fig. 2a)^23^. As expected, DT did not alter cell numbers in WT mice, but significantly depleted HSPCs (KLS, Fig. 2b; and MyPro, Fig. 2c; Supplementary Fig. 1a) and mature (GM, B, and T) cells (Fig. 2d) in the BM of Vav-DTR mice, similar to levels of ablation achieved with irradiation. Thus, DT very rapidly depleted the vast majority of all hematopoietic cells in the BM of Vav-DTR mice.

**Figure 3:**
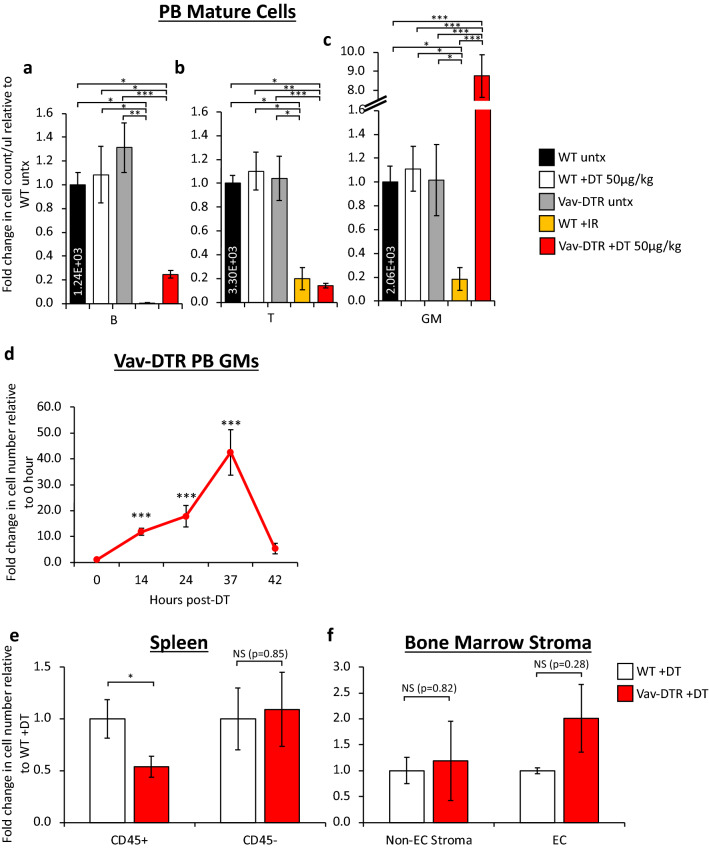
Cells in the PB and spleen of Vav-DTR mice are differentially affected by DT in vivo*.* Treatment groups are indicated in Fig. 2A. DT (50 µg/kg) depleted B (**a**) and T (**b**) in the peripheral blood of Vav-DTR mice (red bar) at 24 h post-treatment, similar to 9-days post-sublethal (750 rads) irradiation (IR, yellow bar). (**c**) GMs increased in the peripheral blood of Vav-DTR mice at 24 h post-DT treatment, but were depleted by irradiation. (**a-c**) WT mice were unaffected by DT treatment (white bars), with cell numbers similar to untreated WT mice (black bar) and untreated Vav-DTR mice (gray bars). The numbers in the black bar represent absolute cell count per microliter of PB. Bar graphs indicate the fold change in cell number relative to WT untreated. N = 6–14 mice in at least three independent experiments. Error bars indicate SEM, *p < 0.05, **p < 0.01, ***p < 0.001 (One-way ANOVA with Tukey post-hoc test). (**d**) Time course of DT effects on PB GMs of Vav-DTR mice. Line graph indicates the fold change in cell number relative to pre-DT time point (0 h), showing an initial increase in GMs until 37 h post-DT followed by quick depletion by 42 h. Later time points could not be collected due to poor mouse health. N = 2 mice. Error bars indicate SEM, ***p < 0.001 (One-way ANOVA with Dunnett’s post-hoc test). (**e**) Reduction in the number of hematopoietic cells (CD45^+^), but not non-hematopoietic (CD45^−^), cells in the spleen of Vav-DTR mice 24 h after DT (50 µg/kg) treatment (red bars). (**f**) Stromal cells (non-EC stroma; Ter119^−^CD45^−^Sca1^+^CD31^−^) and endothelial cells (EC; Ter^−^CD45^−^Sca1^+^CD31^+^) from the BM of Vav-DTR mice (red bars) were not depleted by DT. Bar graphs indicate the fold change in cell number in Vav-DTR + DT (red bars) relative to WT + DT (white bars) mice. For (**e**,**f**), N = 6–8 mice in at least three independent experiments. Error bars indicate SEM, *p < 0.05 (Student’s t-test). NS, not significant.

In PB, not all mature cells were ablated equally. As expected, B cells (Fig. 3a) and T cells (Fig. 3b) were significantly depleted from Vav-DTR mice 24 h post-DT treatment to levels similar to 9 days post-irradiation. DT-induced depletion of platelets was also observed in Vav-DTR mice (Supplementary Fig. 1b), while RBCs (Supplementary Fig. 1c) remained unaffected at this timepoint, likely due to the lack of Vav1-driven DTR expression by RBCs themselves and consistent with the Vav-GFP mice we previously described^20^. Surprisingly, GM cell counts significantly increased 24 h after DT treatment which contrasts the significant depletion observed 9 days post-sublethal irradiation (Fig. 3c). A time course tail bleed analysis revealed that this increase in GMs was temporary until 37 h post DT treatment, followed by a subsequent steep decrease 42 h post DT in Vav-DTR mice (Fig. 3d). Due to poor overall health after 42 h, mice were sacrificed, and no further time points were recorded. Of note, DT-mediated depletion of mature blood cells was remarkably robust in both BM (Fig. 2) and PB (Fig. 3).

To test the specificity of DT sensitivity to the hematopoietic compartment, we investigated the effects of DT on non-hematopoietic cells of the spleen and BM 24 h post-DT treatment. DT-dependent cell number decrease was found to be specific to cells labeled by the pan-hematopoietic marker CD45, but did not affect CD45- spleen cells 24 h after treatment (Fig. 3e). Additionally, bones were evaluated for endothelial cell (EC) or non-EC stromal populations. Despite contradicting reports of off-target *vav-*driven labeling of ECs^19,20,26^, ECs from our Vav-DTR mice showed no sensitivity to DT (Fig. 3f). Taken together, our in vitro and in vivo data demonstrate that the Vav-DTR mice are exclusively and specifically sensitive to very rapid and robust hematopoietic cell ablation upon administration of DT.

### DT-mediated hematopoietic ablation increased donor chimerism in transplanted recipients

**Figure 4:**
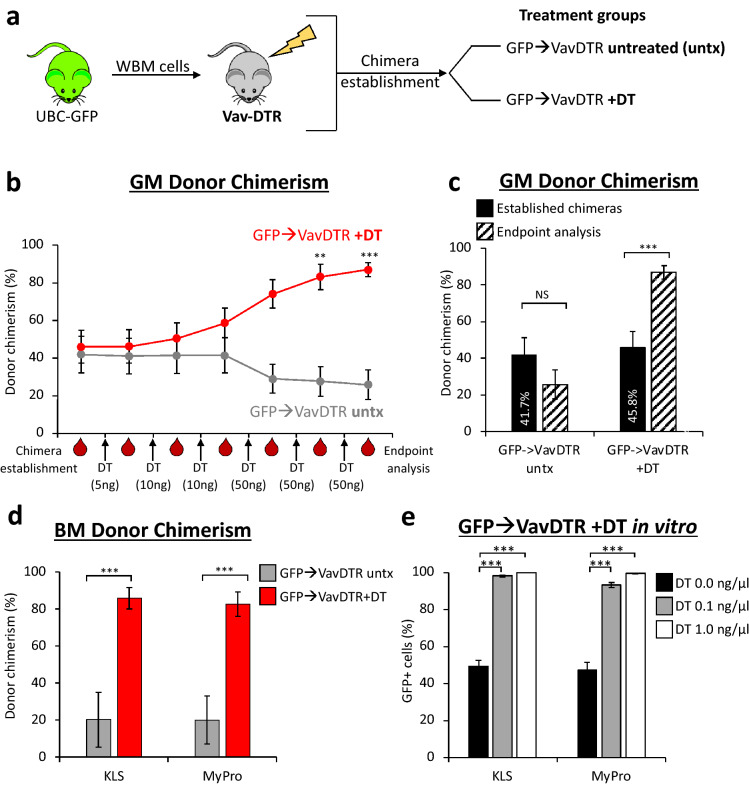
DT treatments selectively increased chimerism of donor-derived WT cells in Vav-DTR recipients. (**a**) Schematic of experimental design. Chimeras were established: UBC-GFP donor BM cells were transplanted into sublethally irradiated Vav-DTR (GFP → VavDTR) recipients. 16 weeks after transplant, these chimeras were split into two groups, a DT treatment (+ DT), and a control untreated (untx). Chimeras in the DT group received multiple (6) doses of DT (5–50 ng), each one week apart and bled intermittently to monitor chimerism levels. (**b**) GM donor chimerism increased over time in GFP → VavDTR chimeras treated with DT but not in the untreated control chimeras. In the graph, the arrows represent DT treatments, and the blood drops represent the tail bleeds performed 1 week after each DT treatment. N = 2–3 mice per group in four independent experiments. Error bars indicate SEM, **p < 0.01, ***p < 0.001 (one-way ANOVA with Dunnett’s post-hoc test). (**c**) Tail bleeds at chimera establishments (prior to DT) and endpoint (following last DT treatment) demonstrated a significantly increase in GM donor chimerism in GFP → VavDTR chimeras treated with DT but not in untreated controls. Bar graphs indicate GM donor chimerism upon chimera establishment (black filled bars) and after DT treatment (endpoint analysis; patterned black bars). The percentages shown in the black bars represent the average donor chimerism at establishment of the chimeras, prior to initiation of the DT regimen. N = 2–3 mice per group in four independent experiments. Error bars indicate SEM, ***p < 0.001 (Student’s t test). *NS* not significant. (**d**) BM KLS and MyPro donor chimerism also significantly increased in GFP → VavDTR + DT chimeras (red bars) compared to untreated GFP → VavDTR controls. N = 2–3 mice per group in four independent experiments. Error bars indicate SEM, ***p < 0.001 (Student’s t-test). (**e**) KLS and MyPro cells sorted from untreated GFP → VavDTR chimeras were treated with DT in vitro. Bar graphs represent the percent of GFP + donor (UBC-GFP) cells. The increase in the proportion of GFP + cells to nearly 100% indicated that host cells (GFP-) were significantly depleted after a 3-day 0.1 ng/µL (gray bar) and 1.0 ng/µL (white bar) DT treatment, while untreated cells maintained the ratio observed in vivo (black bar, DT 0.0 ng/µL). N = 3 in 3 independent experiments. Error bars indicate SEM, ***p < 0.001 (One-way ANOVA with Dunnett’s post-hoc test). *WBM* whole bone marrow, *KLS* ckit^+^Lin^−^Sca1^+^ cells include HSCs and MPPs, *MyPro* myeloid progenitors are c-kit^+^Lin^−^Sca1^−^ cells include CMPs, MEPs, and GMPs, *untx* untreated, *DT* diphtheria toxin.

Having demonstrated the efficiency and specificity of DT in depleting hematopoietic cells in the Vav-DTR mouse model, we hypothesized that this system could be exploited to increase donor chimerism upon transplantation. Thus, we transplanted WBM cells from UBC-GFP mice (where all cells express GFP) into sub-lethally irradiated (non-fluorescent) Vav-DTR mice (GFP → VavDTR; Fig. 4a) or WT (GFP → WT; Supplementary Fig. 2a) mice. After chimeras were established (> 16-weeks post-transplant), we treated them with increasing sequential doses of DT. Since we previously observed how specific, effective, and quick DT-induced cell death occurs (Figs. 2, 3), we reasoned that multiple increasing doses of DT would avoid abrupt and overwhelming cell death in these chimeras. We analyzed the peripheral blood composition 1 week after each DT injection to determine any changes in donor chimerism (Fig. 4b, Supplementary Fig. 2b). As shown in Fig. 4b, we observed a gradual increase in donor chimerism upon DT treatment of the GFP → VavDTR chimeras, which became significant after a second 50 ng DT dose. Meanwhile, donor chimerism in the respective control chimeras (GFP → VavDTR untx) remained stable (Fig. 4b, Supplementary Fig. 2b). When comparing GM donor chimerism at chimera establishment with endpoint analysis, we observed that donor chimerism increased to over 90% in DT-treated GFP → VavDTR chimeras where the host was DT-sensitive (Fig. 4c). Similar to GFP → VavDTR untreated chimeras (Fig. 4b,c), GM donor chimerism remained unaltered in GFP → WT chimeras untreated or DT-treated (Supplementary Fig. 2c). Consistent with GM donor chimerism, total donor chimerism in GFP → VavDTR chimeras increased significantly after DT treatment as well, while no significant differences were observed in all other chimera groups (Supplementary Fig. 2d). DT treatment also led to a significant increase in bone marrow donor chimerism in the GFP → VavDTR chimeras compared to the untreated controls (Fig. 4d). We also isolated KLS and MyPro cells from control GFP → VavDTR chimeras (GFP → VavDTR untx) and treated them in vitro (as in Fig. 1e) to confirm that the results observed in vivo were due to differential DT sensitivity*.* Similar to the in vivo data, GFP + and WT cells from GFP → WT chimeras were not affected by DT in vitro (Supplementary Fig. 2e). In contrast, and as expected, only GFP + donor cells from Vav-DTR recipient mice survived DT treatment while Vav-DTR cells were depleted (Fig. 4e, Supplementary Fig. 2f). Overall, these data demonstrate the specificity of DT in a transplant setting, which allow selective increase of donor chimerism in situ.

### Generation and characterization of an HSC-specific DT-sensitive mouse model

**Figure 5:**
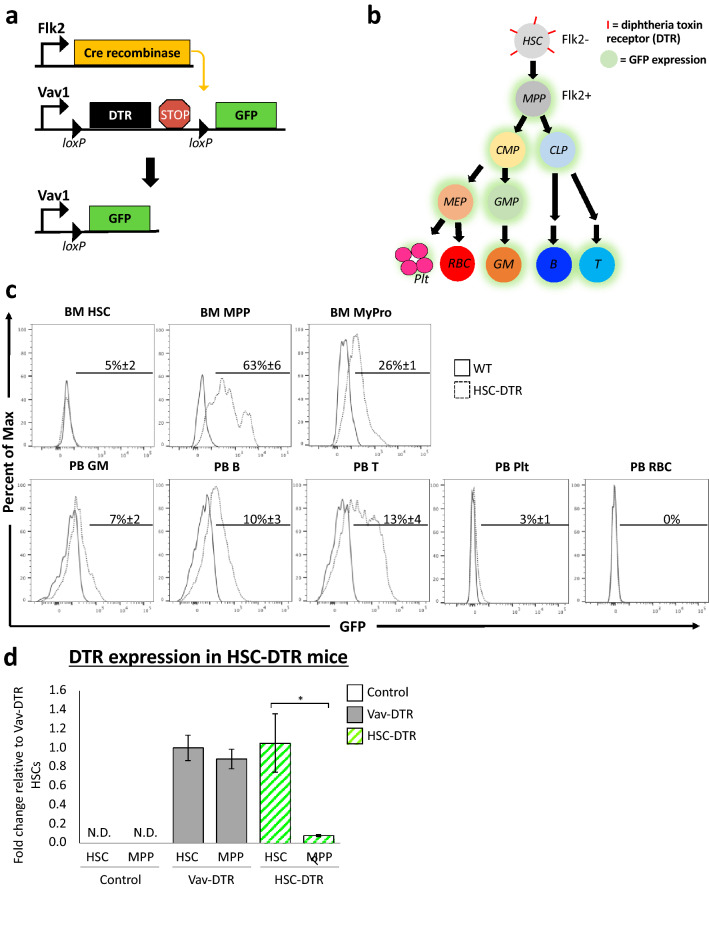
DTR expression is restricted to HSCs in HSC-DTR mice. (**a**) Flk2-Cre mice were crossed to Vav-DTR mice to generate “HSC-DTR” mice. (**b**) Simplified model of hematopoietic tree in HSC-DTR mice. Cell types that are Flk2+ or derived from Flk2+ progenitor cells should express GFP but not DTR, while Flk2− cells that have no history of Flk2 expression should express DTR and remain GFP−. (**c**) Representative histograms of flow cytometry data indicating GFP expression levels in BM and PB cells in WT mice (black line) and HSC-DTR mice (dotted black line). Percentages represent the frequency of cells labeled by GFP. (**d**) RT-qPCR analysis of DTR HSCs and MPPs sorted from control (white bars), Vav-DTR (gray bars), and HSC-DTR (white and green pattern bars) mice revealed that the DTR transgene is efficiently and significantly deleted in MPPs from HSC-DTR mice. Bar graph indicates the relative expression of DTR, normalized to β-actin. N = 6 mice. Error bars indicate SEM. *p < 0.05 (Student’s t-test). *N.D* not detected.

We next crossed our Vav-DTR mouse to the well-characterized Flk2-driven Cre mouse line^27–32^ to generate “HSC-DTR” mice where DTR would be expressed only by HSCs. In this model, Flk2-driven expression of Cre recombinase catalyzes the excision of loxP-flanked transgenes in all hematopoietic cells except HSCs^28,29^ (Fig. 5a). Flk2 is expressed at the MPP stage, thus all cells expressing Flk2 or with a history of Flk2 expression will have undergone loxP recombination. When crossed to the Vav-DTR mice, Flk2-Cre should excise the DTR gene and STOP codon and induce irreversible excision of the DTR transgene and subsequent expression of GFP in all hematopoietic cells except HSCs (Figs. 1a, 5a,b). Treatment of these mice with DT should then lead to HSC-specific cell death.

To evaluate floxing efficiency and the ability of the reporter construct to label hematopoietic cells with GFP fluorescence, HSPCs and mature cell populations were isolated from the BM and PB of HSC-DTR mice. Flow cytometry analysis revealed GFP reporter expression in a fraction of all hematopoietic cells of HSC-DTR, but not WT, mice except for HSCs and circulating red blood cells and platelets (Fig. 5c). We noted that the overall proportion of cells expressing GFP was far from complete, even in the lymphoid lineage that expresses robust levels of the Flk2-Cre transgene at multiple stages of differentiation^28–34^. Although both the frequency of GFP+ cells and GFP expression levels were low, we detected significantly reduced levels of the DTR transgene in (GFP+) MPPs and compared to (GFP−) HSCs from HSC-DTR mice (Fig. 5d). Thus, it appeared that the DTR transgene was deleted as intended, and that GFP expression was insufficiently strong to be a reliable indication of Flk2-Cre recombination (floxing) efficiency in this model.

### In vitro and in vivo DT sensitivity is specific to HSCs in HSC-DTR mice

**Figure 6:**
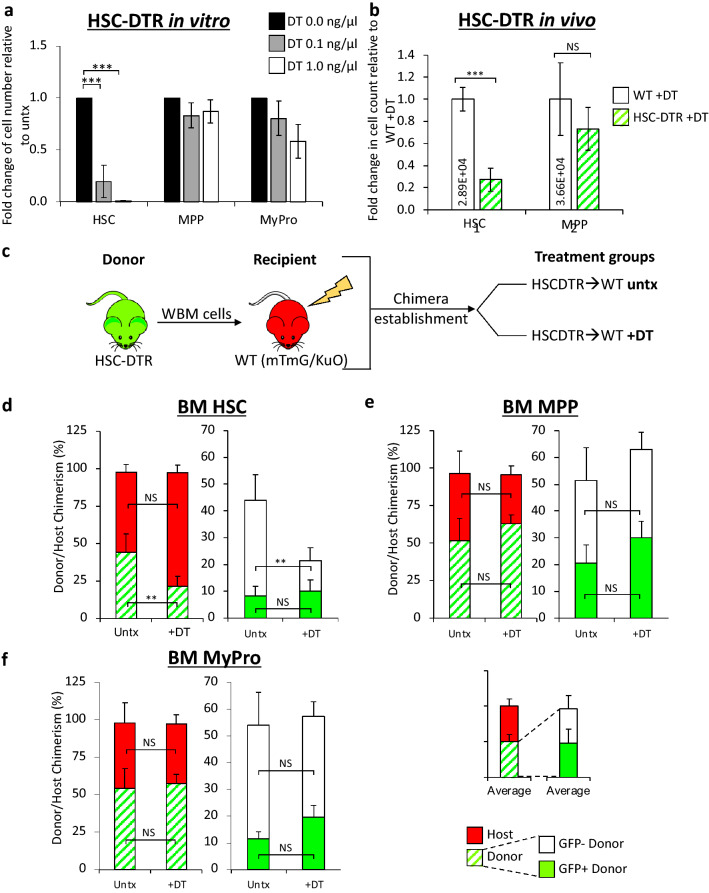
DT selectively depleted HSCs in the HSC-DTR mouse model. (**a**) HSCs isolated from HSC-DTR mice were significantly depleted by DT treatment in vitro, while MPPs and MyPros from the same mice were unaffected. Bar graphs indicate the fold change in cell number relative to untreated (black bar, DT 0.0 ng/µL) upon a 7-day 0.1 ng/µL (gray bar) and 1.0 ng/µL (white bar) DT treatment. N = 3 independent experiments. Error bars indicate SEM, ***p < 0.001 (One-way ANOVA with Dunnett’s post-hoc test). (**b**) HSCs in HSC-DTR mice were significantly depleted by DT (100 ng/mouse) treatment in vivo, while MPPs remained unaffected. Bar graphs indicate the fold change in cell number relative to DT-treated WT mice (white bars). The numbers in the white bars represent absolute cell count in the BM. N = 2–5 mice in three independent experiments. Error bars indicate SEM, ***p < 0.001 (Student’s t-test). (**c**) Schematic of chimera experimental design. WBM cells from HSC-DTR donor mice were transplanted into sublethally irradiated (500 rads) WT recipients (HSCDTR → WT). Upon chimera establishment, chimeras were split into two groups: one was treated with DT and the control group was untreated. Chimeras in the DT group received one dose of 100 ng DT, 24 h prior to takedown for BM analysis. (**d**) HSC donor chimerism significantly decreased upon DT treatment, while (**e**) MPP and (**f**) MyPro donor chimerism remained unaffected. The first set of bar graphs for each cell type represents donor (white and green pattern) and host (red) chimerism, while the second set of bar graphs shows the breakdown of GFP- (white) and GFP + (green) donor chimerism. N = 1–3 mice in 4 independent experiments. Error bars indicate SEM, **p < 0.01 (Student’s t-test).

We then tested whether DT sensitivity was indeed limited to the HSC population. We sorted HSCs, MPPs, and MyPros and treated them with DT in vitro. Consistent with the weak GFP expression in this model (Fig. 5c), the MPP and MyPro populations included both GFP+ and GFP− cells. As expected, HSCs, but not MPPs and MyPros, were efficiently depleted by two different doses of DT (Fig. 6a, Supplemental Fig. 3a). We additionally treated HSC-DTR mice with DT in vivo and observed a significant reduction of HSCs in HSC-DTR mice, while MPPs remained unaffected (Fig. 6b). These data indicated that the DTR gene had been successfully excised in HSC progeny to make these cells DT-resistant. In contrast, HSCs remained highly DT sensitive, consistent with the floxing pattern of previous Flk2-Cre models^20,28,29,33^.

We next transplanted WBM cells from HSC-DTR mice into sub-lethally irradiated fluorescent WT mice (HSCDTR → WT) to establish chimeras (Fig. 6c). Importantly, the mTmG or KuO fluorescent hosts uniformly and robustly express their respective transgene, allowing identification of both GFP + and GFP- donor cells. After recovery and verification of chimerism (Supplementary Fig. 3b), we treated these chimeras with a single dose of DT 24 h prior to analysis of BM donor chimerism (Fig. 6c–f). This analysis revealed that DT significantly reduced HSC donor chimerism by specifically killing donor GFP- HSCs (Fig. 6d, green patterned bars). Importantly, the percentage of donor MPPs (whether GFP+ or GFP−) (Fig. 6e) and MyPros (GFP+ and GFP−; Fig. 6f) were unaffected by DT treatment in vivo. Overall, these in vitro and in vivo data demonstrate that DT selectively targets HSCs in HSC-DTR mice and suggests that the HSC-DTR mouse line is a suitable model for *in vivo* targeted ablation of HSCs.

## Discussion

We have developed two new mouse models where cell death of either nucleated hematopoietic cells, or only HSCs, can be induced in vivo by administration of DT. The “Vav-DTR” mice show Vav1-driven expression of DTR in all hematopoietic cells (Fig. 1). This Vav-dependent model is consistent with the previously reported hematopoietic specificity of Vav1 activity^19–21^. Our in vitro (Fig. 1) and in vivo (Figs. 2, 3) data show that DT selectively and efficiently ablates hematopoietic cells from Vav-DTR mice. Interestingly, we also observed a transient increase in PB GMs suggesting a neutrophilic influx to possibly remove cellular debris accumulated from extensive cell death upon systemic DT treatment^35–37^. Similarly, lower levels of splenic mature cell depletion after DT treatment compared to BM and PB may be due to transient neutrophil influx into the spleen as well. More importantly, non-hematopoietic cells from the spleen and BM stromal cells of Vav-DTR mice remained unaffected by DT (Fig. 3e,f). We speculate that the trend towards an increase in ECs in the Vav-DTR BM upon DT treatment, which was previously observed by others in a similar context^36^, is more likely due to increased recovery, rather than an increase in actual cell numbers, of ECs due to decreased adherens to BM stroma upon the quick and overwhelming DT-induced death of hematopoietic BM cells. We also demonstrated that the selectivity of DT sensitivity could be exploited in a transplant setting to increase donor chimerism (Fig. 4).

Given how quickly, efficiently, and specifically DT leads to death of DTR-expressing cells, DT pre-conditioning in the Vav-DTR model must be more carefully optimized before use as an alternative to irradiation. The massive death of DTR-expressing cells withing 24 h of in vivo administration of 50 µg/kg DT may cause death due to vaso-occlusion and/or inability of rescue by transplanted cells that cannot immediately replenish host cells. Two straightforward options that we have not yet been able to fully explore is to reduce the DT dose and/or utilize HSC-DTR mice as recipients. A third alternative was uncovered by a recent publication that employed an inducible Gata2 knockout model for the depletion of HSCs. The study demonstrated that HSPCs transplanted into unconditioned recipients persist in the BM for at least 4 weeks, allowing for post-transplant niche clearance and subsequent reconstitution of the pre-transplanted HSPCs^38^. This intriguing result suggests that a post-transplant conditioning approach may address the timing discrepancy between DT-induced host cell death and rescue by donor cells, thus making our Vav-DTR and/or HSC-DTR models potentially suitable for HSC engraftment in a non-irradiated, more selectively perturbed environment.

We then crossed the Vav-DTR mice to our well-characterized Flk2-Cre transgenic mice to achieve HSC-specific DTR expression (Fig. 5). We previously demonstrated efficient Flk2-Cre-mediated excision of a floxed transgene in all hematopoietic cells except for HSCs^28–32^. In the new “HSC-DTR” model, HSCs express the DTR while all cells downstream of HSCs, via differentiation through Flk2+ MPPs, do not express the DTR (Fig. 5d) and were therefore unaffected by DT (Fig. 6). Although GFP expression in this model is relatively low and underestimates floxing efficiency, our in vitro and in vivo data demonstrated that DT-sensitivity was indeed highly restricted to the HSC compartment of HSC-DTR mice (Fig. 6).

These two new mouse models are suited to investigate the cellular mechanisms of hematopoietic homeostasis*, *in situ HSC differentiation cascades, the ability of progenitor cells to sustain hematopoiesis in the absence of HSCs, and to manipulate post-transplant engraftment and chimerism similar to a recently published study^38^. Experimental use of these mice has the potential to uncouple self-renewal capability in situ from the ability to provide long-term hematopoietic reconstitution upon transplantation and may therefore impact our understanding of the mechanisms regulating self-renewal. For example, these mice could be utilized to ask such questions as: are multipotent progenitors capable of self-renewal in situ, despite their inability to self-renew upon transplantation? Is differentiation re-routed to cells necessary for survival, at the expense of other cell types, when endogenous HSCs are ablated? Certainly, transplantation assays have demonstrated the ability of HSCs to self-renew and differentiate into all the hematopoietic lineages^23,24,33,39–41^. However, the extent to which this reflects in situ hematopoiesis is unclear as transplantation is conducted under broadly damaging conditioning regimens that force HSCs to proliferate to replenish the entire hematopoietic system of a recipient mouse^42–44^. Importantly, recent studies have also argued that the in situ contribution of HSCs to steady-state hematopoiesis is less than what is observed upon transplantation^36,42–44^. The use of our new HSC-DTR mice, where a large proportion of HSCs can be depleted due to expression of DTR, could therefore complement these studies, including a recent functional report suggesting that hematopoiesis may proceed normally despite a reduction of HSCs to less than 10% of normal numbers^36^.

Here, we generated two new mouse models, Vav-DTR and HSC-DTR, which respectively achieve efficient and selective depletion of all hematopoietic cells or only HSCs in response to DT treatment. Both mouse models were extensively characterized and showed restricted DTR expression in selected tissues or cells of interest, along with specific DT sensitivity in vitro, in vivo, and in transplantation settings. These two new mouse models will be useful tools to advance our understanding of hematopoietic homeostasis, HSC engraftment, and properties of HSCs under steady-state and varying physiological conditions.

## Methods

### Mice

Vav-DTR and HSC-DTR mice were generated in house as described below. WT C57BL/6 (cat# 000664), WT UBC-GFP (cat# 004353), WT mT/mG (cat# 007576) were purchased from Jackson Laboratories. Mice were maintained and bred in the UCSC AAALAC-approved vivarium according to IACUC approved protocols, under which all experiments were conducted. In addition to this, we confirm that the experimental protocols, Forsc1906, were approved by the UCSC IACUC (Institutional Animal Care and Use Committee), which is a named institutional and/or licensing committee. Mice were sacrificed by CO_2_ (carbon dioxide) inhalation, as per our IACUC-approved protocols, Forsc1906. The study was carried out in compliance with the ARRIVE guidelines.

### Generation of Vav-DTR and HSC-DTR transgenic mice

The Vav-DTR plasmid was generated by inserting the DTR sequence followed by a STOP codon between loxP sites, flanked at the two ends by Vav regulatory elements and a GFP sequence respectively. The vector was linearized and injected into pronuclei of C57BL/6 mice at the University of California Santa Cruz (UCSC) transgenic facility. Multiple founders were used to establish a colony, but founder lines were not analyzed separately. Characterization of the founders revealed nothing of concern and consistent normal phenotypes. Vav-DTR litters were genotyped using the following primers: 5′-AGCTGCTCCAGGCTCTCG-3′ (binds to DTR sequence) and 5′-GTGTTGTAGTTGTCCCCACTGG-3′ (binds to Vav1 regulatory elements sequence). HSC-DTR mice were generated by breeding Vav-DTR mice and Flk2-Cre mice. The PB from male HSC-DTR mice was analyzed by flow cytometry to confirm Flk2-Cre recombinase activity and determine Cre-driven DTR excision, referred to as “floxing”, efficiency. Floxing efficiencies ranged depending on the cell type. Only male HSC-DTR mice were analyzed as Flk2-Cre recombination is inefficient in females^28,30,33^.

### qPCR analysis

DNA was isolated from BM cells sorted from WT and Vav-DTR mice using QIAamp^®^ DNA Blood Mini Kit (Qiagen) according to manufacturer’s protocol (Fig. 1d). qPCR was run on a QuantStudio 6 Flex PCR thermal cycler (Thermo Fisher Scientific) using SensiMix™ SYBR^®^ No-ROX Kit (Bioline) according to the manufacturer’s protocol. Messenger RNA was extracted from the various tissues using Trizol (Invitrogen). RNA was used to obtain cDNA using High Capacity cDNA Reverse Transcriptase Kit (Applied Biosystems) according to the manufacturer's protocol (Fig. 5d). Quantitative real-time PCR was run on a ViiA 7 or QuantStudio 6 Flex PCR thermal cycler (Thermo Fisher Scientific) using SensiMix SYBR No-ROX Kit (Bioline) according to the manufacturer's protocol. β-actin was used to normalize expression levels. qPCR was conducted using the following primers: 5′-AGGCAAGGGACTAGGGAAGA-3′ and 5′-CCACCACAGCCAGGATAGTT-3′ for DTR; 5′-CCACAGCTGAGAGGGAAATC-3′ and 5′-CTTCTCCAGGGAGGAAGAGG-3′ for β-actin.

### Flow cytometry

BM and spleen cells were obtained by crushing the tibia and femur or spleen in 1X PBS supplemented with 5 mM EDTA with 2% serum. PB was collected directly into 1X PBS supplemented with 5 mM EDTA with 2% serum from the tail vein or femoral artery. Single cell suspensions were passed through 70-micron filters, and RBCs were lysed (spleen and PB only). Cells were then stained with monoclonal antibodies on ice in the dark for 20 min and analyzed using a FACSAria or an LSRII flow cytometer (BD Biosciences, San Jose, CA) as described previously^23,31,45^. FlowJo Software 10.7.1 (Ashland, OR) was used for data analysis and display. Live cells were determined by staining with propidium iodide. Following pre-gating on single, live cells, hematopoietic cell populations were defined by the following cell surface phenotypes: KLS (Lin^–^Sca1^+^c-kit^+^), HSCs (Lin^–^Sca1^+^c-kit^+^Slam^+^Flk2^−^), MPPs (Lin^−^Sca1^+^c-kit^+^Slam^–^Flk2^+^), MyPros (Lin^–^Sca1^–^c-kit^+^), GMs (Ter119^−^CD3^−^B220^−^Mac1^+^Gr1^+^), T cells (Ter119^−^Mac1^−^Gr1^−^B220^−^CD3^+^), B cells (Ter119^−^Mac1^−^Gr1^−^CD3^−^B220^+^), platelets (FSC^lo^Ter119^−^CD61^+^), and RBCs (Ter119^+^). The lineage (Lin) mixture consisted of antibodies recognizing CD3, CD4, CD5, CD8, B220, Gr1, Mac1, and Ter119 cell surface proteins. Bone endothelial cells (ECs; CD45^–^Ter119^–^CD31^+^Sca^+^) and non-EC stroma cells (CD45^–^Ter119^–^CD31^−^) were prepared as described previously^46^. Briefly, tibia and femur were dissected and homogenized with PBS using a mortar and pestle. Bone fragments were digested in a 3 mg/mL collagenase I solution for 1 h at 37 °C with intermittent vortexing and finally neutralized by adding serum containing EDTA/PBS media. Samples were then washed with PBS, filtered, and stained prior to analysis by flow cytometry.

### Cell sorting

Hematopoietic cells were isolated and prepared from the BM of mice in accordance with UCSC guideline as described above and previously using a BD FACSAria^23,24,28,30,33,46–50^.

### Absolute cell number quantification

A known volume of PB was mixed with an antibody solution in 1X PBS supplemented with 5 mM EDTA with 2% serum containing a known quantity of Calibrite APC beads prior to flow cytometry analysis. For tissues, such as BM and spleen, a known quantity of beads was added to each tissue prior to homogenization. The ratio of number of beads added to the sample to the number of beads collected by flow cytometry was used to calculate the absolute number of mature cells per microliter of blood or within each tissue^23,49^.

### Irradiation assays

Mice were irradiated using an X-ray tube irradiator (Faxitron CP-160). For the experiments described in Figs. 2 and 3, PB and BM cells were analyzed by flow cytometry 9 days after sub-lethal (~ 750 rads) irradiation as this time point represents the lowest detectable cell numbers post-irradiation, prior to recovery^23^. Sublethal irradiation is often used as the preferred conditioning regimen of host mice prior to transplant.

### Transplantation assays

Transplantation assays were performed as previously described^23–25,28,30,33,47^. For the Vav-DTR chimeras, 3.75 million or 7.5 million whole bone marrow (WBM) cells from donor UBC-GFP mice were retro-orbitally transplanted into ¾ (~ 750 rads) or ½ (~ 500 rads) lethally irradiated Vav-DTR and WT hosts. These two chimera set ups, as expected, led to similar donor chimerism to allow comparison of experiments. For the HSC-DTR chimeras, 1 million WBM from donor HSC-DTR mice were retro-orbitally transplanted into sub-lethally irradiated (~ 500 rads) mTmG or KuO hosts. Recipient mice were bled at 4-, 12-, and 16-weeks post-transplantation via the tail vein for analysis of donor/host contribution in the peripheral blood (data not shown), detectable by GFP or Tomato/KuO expression, to confirm long term multilineage reconstitution.

### Diphtheria toxin treatment

DT (50 µg/kg; Sigma) was administered to WT and Vav-DTR mice via intraperitoneal (i.p.) injection 24 h prior to take-down to determine depletion of hematopoietic or non-hematopoietic cells as shown in Figs. 2 and 3. BM chimeras generated with GFP donor cells into Vav-DTR (GFP → VavDTR) or control WT (GFP → WT) were administered 6 DT doses ranging between 5 and 50 ng/mouse and bled 1 week after each DT treatment as shown in Fig. 4b. HSC-DTR mice were administered 100 ng (~ 5 µg/kg) of DT 24 h prior to BM analysis as shown in Fig. 6b. BM chimeras, with HSC-DTR donor cells into WT recipients (HSCDTR → WT) were administered 100 ng/mouse (~ 5 µg/kg) of DT 24 h prior to takedown for BM analysis. 0.1 ng/µL or 1.0 ng/µL DT was added once to the cell culture media for 7 (Figs. 1e,f, 6a, Supplementary Fig. 3a) or 3 (Fig. 4e, Supplementary Fig. 2e,f) days prior to analysis by flow cytometry to determine in vitro DT sensitivity of Vav-DTR and HSC-DTR cells.

### In vitro culture

Using anti-CD117/cKit microbeads (Miltenyi Biotec), BM cells from WT, HSC-DTR, Vav-DTR, and chimeric mice were enriched for c-Kit positive cells and sorted via flow cytometry into 5 mM EDTA in 1X PBS with 2% serum and then spun down. 100 HSCs, 200 MPPs, and 500 MyPros were plated in triplicates in IMDM media supplemented with 20% FBS, TPO (50 ng/mL), SCF (50 ng/mL), IL-6 (20 ng/mL), IL-3 (10 ng), IL-11 (20 ng/mL), 1X Primocin, and 1X Non-Essential Amino Acids. Live and nucleated cells were harvested and analyzed by flow cytometry after 7 days for Figs. 1e,f, 6a and Supplementary Fig. 3a. HSCs, MPPs, and MyPros were analyzed after 3 days in culture for Fig. 4e and Supplementary Fig. 2e,f.

### Statistics

Unpaired two-tailed Student’s t-tests and one-way ANOVAs adjusted for multiple comparisons with Tukey or Dunnett’s post-hoc tests were used to assess statistical significance for comparisons of different groups, as appropriate. The sample size, number of independent experiments, and p values are provided for each experiment in the respective figure legend.

## Supplementary Information

Supplementary Information.

## Data Availability

No datasets were generated or analyzed during the current study.
