# Supplementary material for: New transgenic mouse models enabling pan-hematopoietic or selective hematopoietic stem cell depletion in vivo
Source: Sci Rep. 2022 Feb 24;12:3156. doi: 10.1038/s41598-022-07041-6 (PMC8873235; doi:10.1038/s41598-022-07041-6)

New transgenic Mouse Models Enabling Pan-Hematopoietic or Selective Hematopoietic Stem Cell Depletion *In Vivo*

Alessandra Rodriguez y Baena[1,2], Smrithi Rajendiran[1,3], Bryce A. Manso[1,3], Jana Krietsch[1,3], Scott W. Boyer[1,2], Jessica Kirschmann[1], E. Camilla Forsberg[1,3*]

[1]Institute for the Biology of Stem Cells, University of California-Santa Cruz, Santa Cruz, CA 95064, USA

[2]Program in Biomedical Sciences and Engineering, Department of Molecular, Cell, and Developmental Biology, University of California-Santa Cruz, Santa Cruz, CA 95064, USA

[3]Biomolecular Engineering, University of California-Santa Cruz, Santa Cruz, CA 95064, USA

*Correspondence: cforsber@ucsc.edu

Supplementary Figure 1. Platelets from Vav-DTR mice are depleted by DT *in vivo* but red blood cells remain unaffected.

(a) Representative flow cytometry plots from Figure 2b-c showing the MyPro and KLS gates for the five different conditions tested: WT untx, WT +DT, Vav-DTR untx, WT +DT, WT +IR, and Vav-DTR +DT. Pre-gates: Nucleated cells, Live, Lin-.

(b) DT (50 µg/kg) significantly depleted plts in the peripheral blood of Vav-DTR mice (red bar) at 24 hours post-treatment.

(c) RBCs from Vav-DTR mice were unaffected by 24 hrs of DT treatment. **(a-b)** WT mice were unaffected by DT treatment (white bars), with cell numbers similar to untreated WT mice (black bar) and untreated Vav-DTR mice (gray bars). The numbers in the black bar represent absolute cell count per microliter of PB. Bar graphs indicate the fold change in cell number relative to WT untreated. N=6-14 mice in at least three independent experiments. Error bars indicate SEM, *$p<0.05$, **$p<0.01$, ***$p<0.001$ (One-way ANOVA with Tukey post-hoc test).

Supplementary Figure 2. DT treatments did not affect chimerism in non-DTR expressing chimeras.

(a) Schematic of experimental design. Control chimeras established: UBC-GFP donor BM cells were transplanted into sublethally irradiated WT (GFP→WT) recipients. Chimeras were established and treated as in Figure 4.

(b) Flow cytometry plots representing GM donor chimerism (shown as percentages of total GMs) for the different treatment groups at endpoint analysis 1 week after the last dose of DT. Pre-gates: Nucleated cells, Live, $B220^-CD3^-Gr1^+Mac1^+$.

(c) GM donor chimerism remained unaffected in GFP→WT chimeras treated with DT, similar to its respective GFP→WT controls. Bar graphs indicate donor chimerism upon chimera establishment (black filled bars) and after DT treatment (endpoint analysis; patterned black bars). NS, not significant.

(d) Total donor chimerism significantly increased only in GFP→VavDTR chimeras treated with DT but not in GFP→WT chimeras treated with DT. Bar graphs indicate donor chimerism upon chimera establishment (black filled bars) and after DT treatment (endpoint analysis; patterned black bars). N=2-3 mice in four independent experiments. Error bars indicate SEM, *p<0.05 (Student's t-test). NS, not significant.

(e) KLS and MyPro cells sorted from untreated GFP→WT control chimeras were treated for 3 days with two different doses (0.1 ng/ul and 1.0 ng/ul) of DT *in vitro*. Bar graphs represent the percent of GFP+ and WT cells. Donor (UBC-GFP; GFP+) and host (WT; GFP-) cells were unaffected. N=3 in 3 independent experiments. Error bars indicate SEM.

(f) Alternative representation of Figure 4e. Bar graphs represent the percent of GFP+ (UBC-GFP; green bars) and GFP- (Vav-DTR; gray bars) cells.

Supplementary Figure 3. HSCs from HSC-DTR mice were uniquely sensitive to DT.

(a) Representative cell count data of 1 of 3 experiments used to calculate fold change of cell number in Figure 6A. Bar graphs indicate the total live, nucleated, cell number in untreated (black bar, DT 0.0 ng/μl) or DT treated cells, 0.1 ng/μl (gray bar) and 1.0 ng/μl (white bar), 7-days after treatment i*n vitro*. N=3 replicates for condition. Error bars indicate SEM, ***p<0.001 (One-way ANOVA with Dunnett's post-hoc test).

(b) GM host/donor chimerism in HSC-DTR→WT chimeras, 16 weeks post-transplant at chimera establishments. These chimeras were remained untreated until establishment and then were split into an Untx and a +DT group to collect data shown in Figure 6c-f. The first set of bar graphs represents donor (white and green pattern) and host (red) chimerism, while the second set of bar graphs shows the breakdown of GFP- (white) and GFP+ (green) donor chimerism. N=1-3 mice in 4 independent experiments. Error bars indicate SEM. NS, not significant.

Supplementary Figure 1

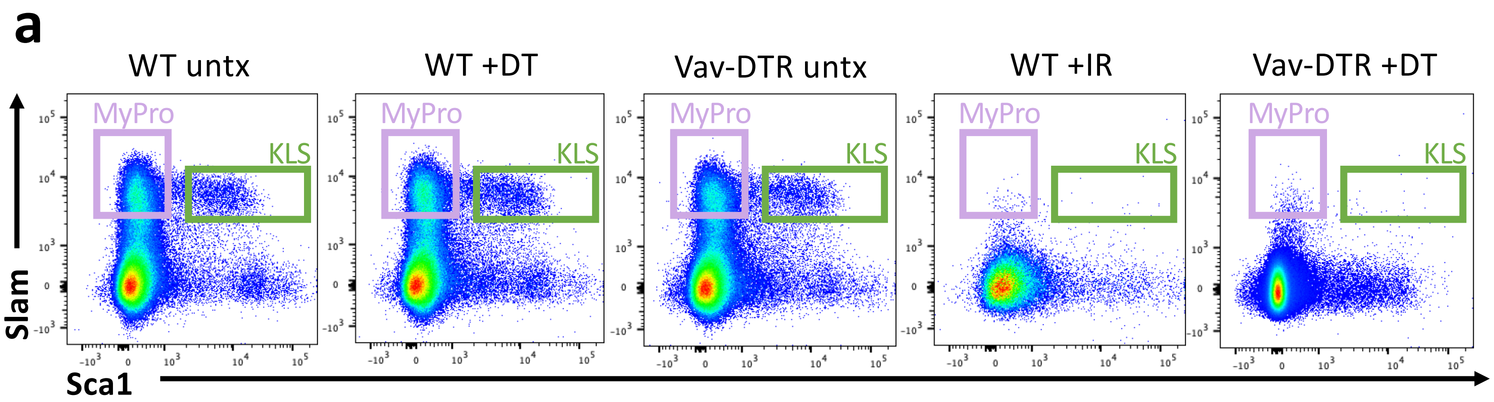

Whole Blood Cells

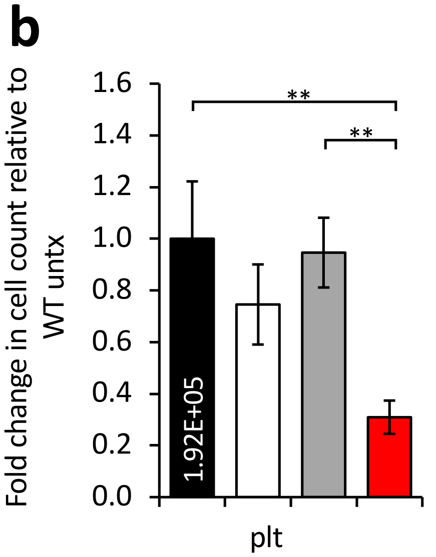

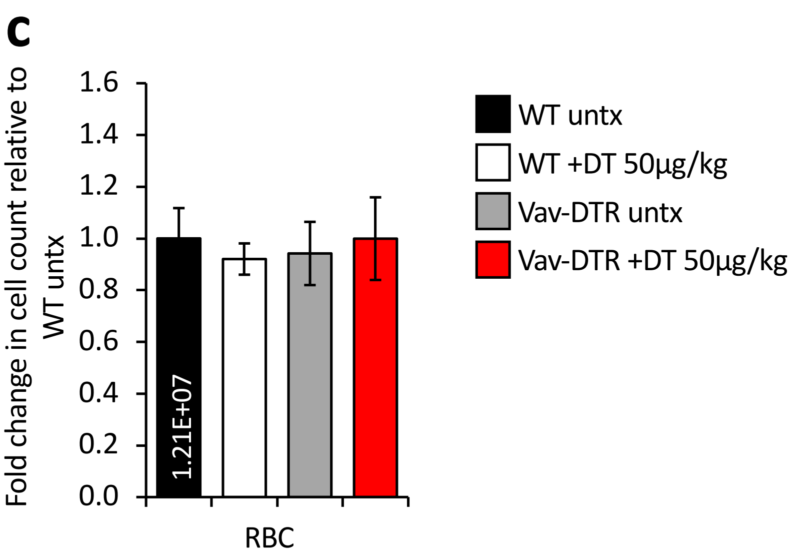

Supplementary Figure 2

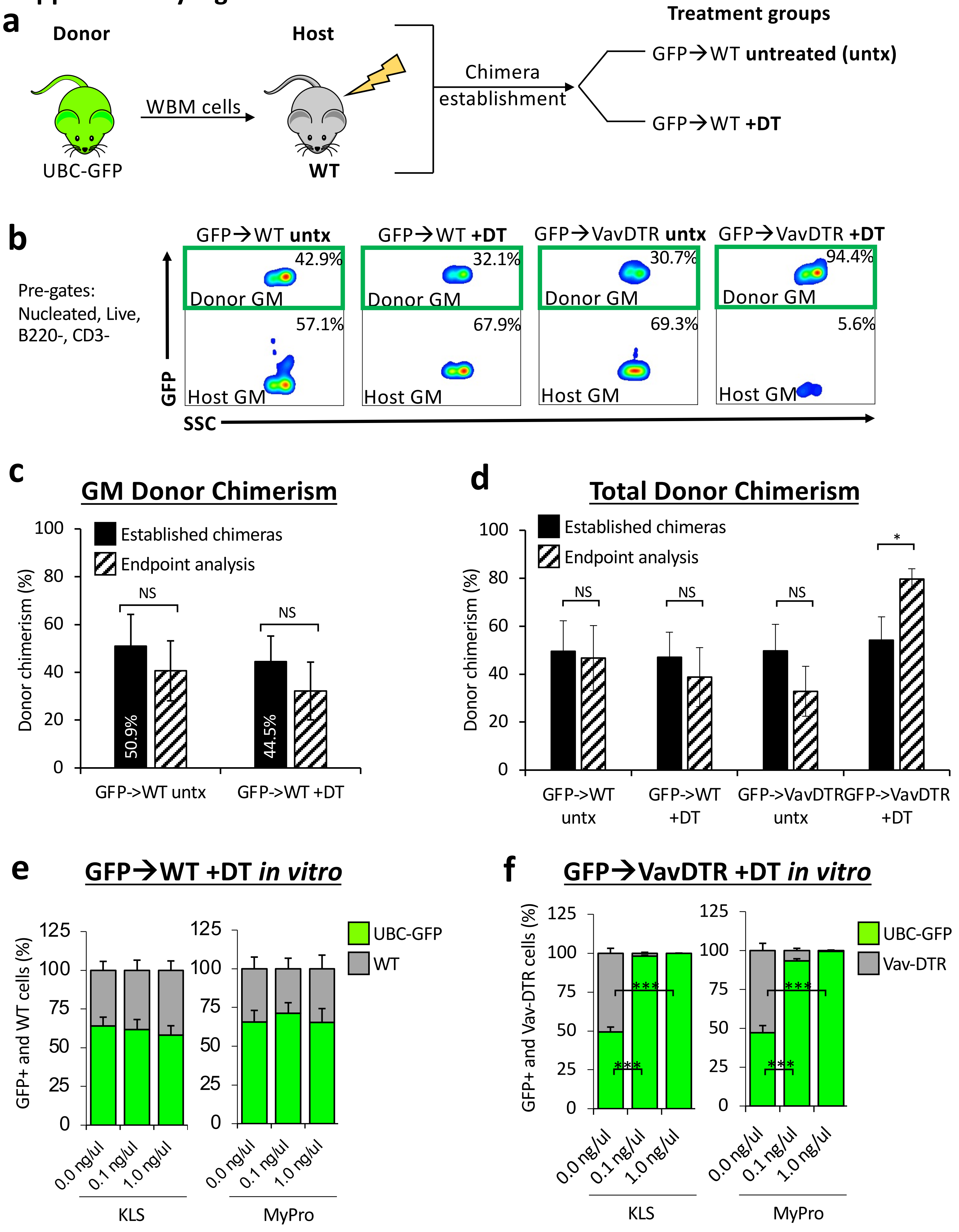

Supplementary Figure 3

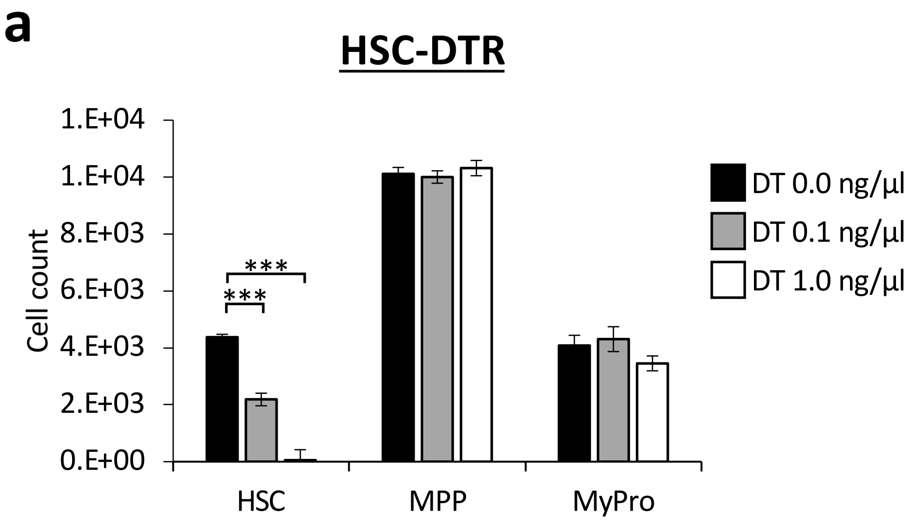

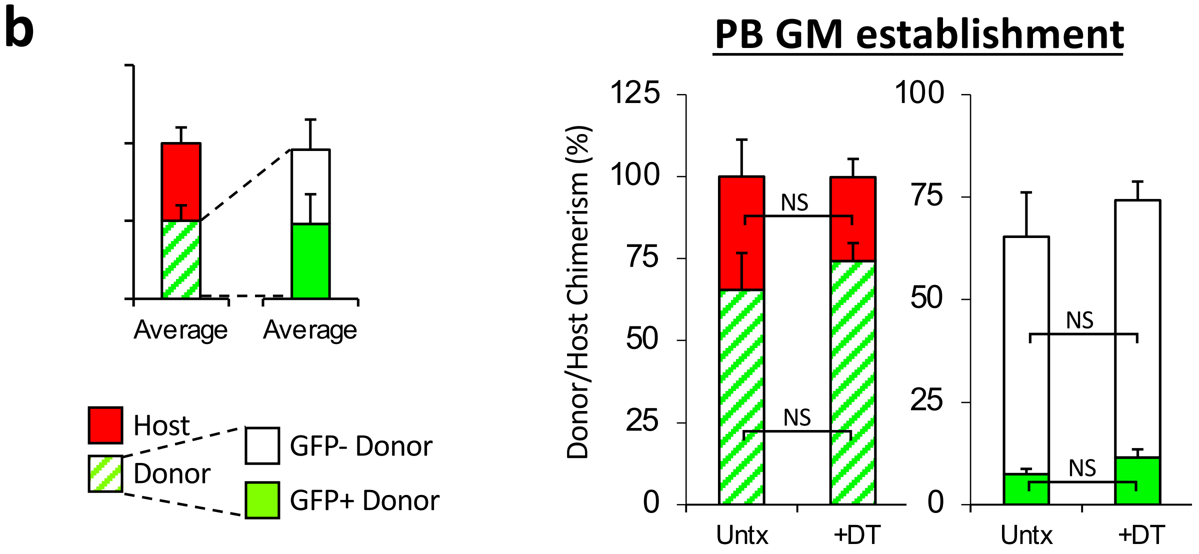

Supplement: Supplementary file 1 — Supplementary Information. [file 41598_2022_7041_MOESM1_ESM.pdf]
